# Cytoprotective Antioxidant, Anti-Inflammatory, and Antifibrotic Impact of Celery Seed Oil and Manuka Honey Against Cyclophosphamide-Induced Cystitis in Rabbits

**DOI:** 10.1155/2022/2863023

**Published:** 2022-03-17

**Authors:** Ayman M. Mousa, Khaled S. Allemailem, Fahad A. Alhumaydhi, Faris Alrumaihi, Ahmad Almatroudi, Mohammad Aljasir, Ameen S. S. Alwashmi, Osamah Al Rugaie, Khaled E. A. Soliman, Abdullah S. M. Aljohani, Waleed Al Abdulmonem, Ahmed A. Ahmed, Arif Khan, Masood A. Khan, Naif AlSuhaymi, Mahdi H. Alsugoor, Wafa Abdullah Al-Megrin, Abulmaaty M. Elsayed

**Affiliations:** ^1^Department of Basic Health Sciences, College of Applied Medical Sciences, Qassim University, Buraydah 51452, Saudi Arabia; ^2^Department of Histology and Cell Biology, Faculty of Medicine, Benha University, Benha 13518, Egypt; ^3^Department of Medical Laboratories, College of Applied Medical Sciences, Qassim University, Buraydah 51452, Saudi Arabia; ^4^Department of Basic Medical Sciences, College of Medicine and Medical Sciences, Qassim University, Unaizah 51452, Saudi Arabia; ^5^Department of Forensic Medicine and Clinical Toxicology, Faculty of Medicine, Sohag University, Sohag 82524, Egypt; ^6^Department of Veterinary Medicine, College of Agricultural and Veterinary Medicine, Qassim University, Buraydah 51452, Saudi Arabia; ^7^Department of Pathology, College of Medicine, Qassim University, Buraydah 51452, Saudi Arabia; ^8^Research Center, College of Medicine, Qassim University, Buraidah 51452, Saudi Arabia; ^9^Department of Emergency Medical Services, Faculty of Health Sciences, AlQunfudah, Umm Al-Qura University, Makkah 21912, Saudi Arabia; ^10^Department of Biology, Faculty of Science, Princess Nourah Bint Abdulrahman University, Riyadh 11671, Saudi Arabia; ^11^Department of Anatomy and Histology, Faculty of Medicine, Mutah University, Mutah, Jordan; ^12^Department of Anatomy and Embryology, Faculty of Medicine, Benha University, Benha 13518, Egypt

## Abstract

Patients treated with cyclophosphamide (CP) usually suffer from severe hemorrhagic cystitis (HC). Our previous study exhibited that mesna + celery cotherapy partially ameliorated HC. Therefore, there is a substantial need to seek alternative regimens to get complete protection against CP-induced HC. The current study investigated the effects of mesna + celery seed oil (MCSO) or mesna + manuka honey (MMH) cotherapy against CP-induced HC in adult male rabbits. The forty rabbits were divided into four equal groups and treated for three weeks. The control group (G1) received distilled water and the second group (G2) received CP (50 mg/kg/week). The third group (G3) received CP + MCSO (CPMCSO regimen), and the fourth group (G4) received CP + MMH (CPMMH regimen). The urinary bladder (UB) specimens were processed to evaluate UB changes through histopathological, immunohistochemical, ultrastructural, and biochemical investigations. In G2, CP provoked HC features (urothelial necrosis, ulceration, and sloughing), UB fibrosis, and TNF-*α* immunoexpression. Besides, CP reduced the activity of antioxidant enzymes (GPx1, SOD3, and CAT) and elevated the serum levels of NF-*κ*B, TNF-*α*, IL-1B, and IL-6 cytokines in G2 rabbits. In contrast, the CPMMH regimen caused significant increments of UB protection against HC in G4 rabbits compared to the partial protection by the CPMCSO regimen in G3. Therefore, our study indicated for the first time that the novel CPMMH regimen resulted in complete UB protection against CP-induced HC via combined antioxidant, anti-inflammatory, and antifibrotic properties.

## 1. Introduction

Cyclophosphamide (CP) is an effective antineoplastic drug, which unfortunately causes multiple side effects such as hemorrhagic cystitis (HC), delayed wound healing, and nephropathy in CP-treated patients [1]. The incidence of HC in those patients may reach up to 75%, with several symptoms including frequency, dysuria, suprapubic pain, and hematuria. CP is metabolized into acrolein (urotoxic hepatic metabolite) by hepatic microsomal hydroxylation to be excreted by both kidneys into the urinary bladder (UB) [2]. Direct urothelial contact with acrolein plays a significant role in HC pathogenesis, which induces a prominent UB inflammation. Acrolein usually induces urothelial apoptosis, necrosis, and damage with subsequent ulceration of the UB [3]. Increased oxidative stress results from increased reactive oxygen species (ROS) and reactive nitrogen species (RNS) production or decreased antioxidant defense mechanisms [4]. Acrolein induces the production of numerous ROS such as hydrogen peroxide (H_2_O_2_), malondialdehyde (MDA), superoxide (O_2_^−^), and reduced glutathione (GSH). Besides, it enhances the formation of RNS such as peroxynitrite (ONOO^−^) from the combination of nitric oxide (NO) with O₂^−^ in the urothelial cells [5]. Therefore, the administration of CP usually evokes HC via overproduction of the ROS and RNS molecules, which cause UB inflammation. Cellular injury and necrosis of the UB involve several mechanisms, including lipid peroxidation of cellular membranes and DNA damage in the inflammatory areas [6]. Besides, the oxidative stress conditions induce the formation of nuclear factor kappa B (NF-*κ*B), which enhances the transcription of tumor necrosis factor-*α* (TNF-*α*), interleukin-1*β* (IL-1*β*), and IL-6. These proinflammatory cytokines have been involved in HC pathogenesis by oxidizing the polyunsaturated fatty acids and inducing inflammation [7].

Mesna administration directly binds acrolein in the UB lumen and neutralizes it into an inert metabolite (thioether), passing safely in urine without damaging the urothelium [8]. Although it is effective for treating induced HC; however, it cannot wholly eradicate the challenging symptoms of HC in 25% of CP-treated cases [9]. Therefore, it is essential to involve other protective antioxidant agents such as celery seed oil (CSO) or manuka honey (MH) beside mesna to reduce the hazards of induced HC [10, 11]. Numerous studies focused on celery (*Apium graveolens*) and honey as effective antioxidants, which substantially reduced the cellular oxidative damage [12, 13]. Besides, their phytochemicals such as terpenoids, flavonoids, and phenolic acids could suppress the activity of proinflammatory cytokines [14].

Celery emerges as one of the most prestigious edible plants, which elicits the attention of researchers as a safe, cheap, and valid phytochemical vegetable growing mainly around the Mediterranean Sea and in Europe [15]. It belongs to the parsley family and usually acts as an effective remedy against numerous inflammatory diseases such as bronchitis, bronchial asthma, arthritis, and hepatitis [16–18]. At the same time, celery has a substantial role in inhibiting appetite, reducing body weight, and preventing hypertension through its diuretic, antioxidant, and anti-inflammatory properties [12, 19].

In contrast, honey possesses powerful wound healing properties and prevents infections for long periods as it offers antimicrobial activity, immunomodulatory properties, and protection against wound infections [20, 21]. The monofloral MH is derived from the manuka tree and has numerous biological properties, including antioxidant, antimicrobial, and anti-inflammatory activities [22]. Regarding the dominant constituents in MH, there are high levels of flavonoids and phenolic acids (glyoxal, benzoic acid, leptosin, quercetin, and chrysin) [23]. A recent study attributed the beneficial nutritional and antioxidant effects of MH to these bioactive polyphenolics and flavonoids [24]. Besides, MH has a high potent antibacterial activity against *Staphylococcus epidermidis*, *Staphylococcus aureus*, and *Streptococcus pyogenes* due to its potential contents of peptides (abaecin), proteins (royalisin), and lysozymes [25]. Henceforth, MH has been employed as a powerful wound-healing remedy for combating various types of infections, including burns, traumatic wounds, and ulcers [26]. Our aim in the current study was to investigate the effectiveness of two novel regimens (CP + mesna + CSO (CPMCSO regimen) and CP + mesna + MH (CPMMH regimen)) against CP-induced HC in an experimental model of rabbits and to evaluate which one of the proposed regimens is more effective against HC.

## 2. Materials and Methods

### 2.1. Drugs and Chemicals

CP (Cytoxan vials 500 mg) and mesna ampoules (uromitexan 400 mg) were obtained from Baxter Healthcare Company (Illinois, USA). The CSO bottles (30 mL) were purchased from Sameera Fragrance (New Delhi, India). The manufacturer extracted the oil by the steam distillation process. The main isolated natural products from the CSO included limonene, sedanolide, and 3‐n‐butyl phthalide, which give the characteristic odor of celery [27]. The MH bottles (250 gm) were purchased from Airborne Company (Canterbury, New Zealand). The ELISA kits of GPx1 (ABIN774992), SOD3 (ABIN6959756), CAT (ABIN628258), NF-*κ*B (ABIN775386), TNF-*α* (ABIN6574142), IL-1B (ABIN6999391), and IL-6 (ABIN6957175) were purchased from antibodies-online GmbH (Aachen, Germany).

### 2.2. Animals and Study Design

**Figure 1:**
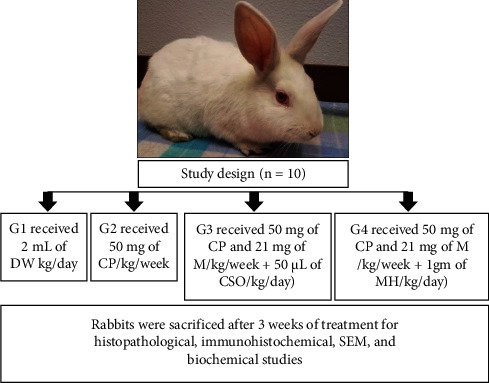
Schematic illustration of the experimental design. The control group (G1) received distilled water (DW), and the second group (G2) received cyclophosphamide (CP). The third group (G3) received CP + mesna (M) + celery seed oil (CSO) = CPMCSO regimen group. The fourth group (G4) received CP + M + manuka honey (MH) = CPMMH regimen group.

Forty adult male, New Zealand rabbits were acclimatized for one week before the commencement of the current experimental study to induce a model of HC. The rabbits' age was 12 weeks, their weight was 1.4–2 kg, and they were fed a standard balanced diet *ad libitum* in metal cages at 24°C. The rabbits were divided into four groups (*n* = 10) and treated for three weeks. The study protocol and procedures were approved by the Institutional Research Ethics Committee of Qassim University (Cams1-2019-2-2-I-5467), conducted following the National Institutes of Health guide for the care and use of laboratory animals (NIH publications no. 8023, revised 1978), and complied with the ARRIVE guidelines [12]. As depicted in Figure 1, the control group (G1) received an orogastric distilled water (2 mL/kg/day), and the second group (G2) received an intraperitoneal injection (IPI) of CP (50 mg/kg/week) to induce HC [11, 28]. The CPMCSO regimen group (G3) received an IPI of CP (50 mg/kg/week) and 21 mg of mesna/kg/week plus 50 *µ*L of CSO/kg/day orally [29–31]. The CPMMH regimen group (G4) received an IPI of CP (50 mg/kg/week) and 21 mg of mesna/kg/week plus 1 gm of MH/kg/day orally [25].

The rabbits were anesthetized with an intramuscular injection of xylazine (6 mg/kg) and ketamine (70 mg/kg) by a professional veterinary doctor to minimize pain, anxiety, and distress effects to the animals. Once the animals became unconscious, a percutaneous intracardiac 19-gauge needle attached to a 20 mL syringe was inserted between the ribs (at the most robust heartbeat) to get blood samples for the various biochemical investigations. Then, the rabbits were euthanized by cervical decapitation to obtain UB specimens for investigating the effectiveness of CPMCSO or CPMMH regimen against CP-induced HC [32]. After ensuring death, the euthanized rabbits were disposed of appropriately following the National Research Council (USA) Guide for the Care and Use of Laboratory Animals [33]. The histopathological (HP), immunohistochemical (IHC), scanning electron microscope (SEM), and biochemical investigations were performed, as described in our previous study [34].

### 2.3. Grading of Macroscopic Hematuria by the Urine Visual Color Test

The visual color scale of the Hemostick test was used to grade the extent of hematuria from 0 to 5 in the urine samples of all groups, as described by Lee et al. [35].

### 2.4. Examining the HP Structure of UB

Small UB specimens from each rabbit were processed to get thin (4 *μ*m) paraffin sections suitable for staining with H&E and Masson trichrome stains, as described by Hussien et al. [36]. The UB general structure was evaluated, and the degree of UB damage (ulceration and sloughing) was rated from 0 to 3 (no, mild, moderate, and severe) by a pathologist who did not know the sequence of groups [12].

### 2.5. Measurement of TNF-*α* Immunoexpression (IE) in the UB

The UB sections were stained by the streptavidin-biotin peroxidase technique to detect the TNF-*α* IE in the UB tissues according to the manufacturer's protocol, as described by Mousa et al. [37].

### 2.6. Examining the UB Ultrastructure

The UB mucosal surface was examined by the SEM to evaluate the degree of UB ulceration and sloughing. Small specimens from each UB were processed to obtain thin sections of UB suitable for SEM examination as described by Poveda et al. [38].

### 2.7. Morphometric Study

“The digital CMOS, TC5PRO camera on a light microscope (Jinan, China) was used to photograph ten fields from the UB sections of each rabbit at magnification 200X to evaluate the UB structure in all groups. Besides, all sections underwent image analysis by ImageJ V1.50i (NHI/USA) to measure the UB ulcer's size and the area percentage of collagen fibers (CFs) deposition and TNF-*α* IE in the UB/10 fields” [12].

### 2.8. Biochemical Measurement of the Antioxidant Enzymes and Proinflammatory Cytokines

The serum levels of antioxidant enzymes (GPx1, SOD3, and CAT) and proinflammatory cytokines (NF-*κ*B, TNF-*α*, IL-1B, and IL-6) were investigated to elucidate the effectiveness of CPMCSO and CPMMH regimens against HC. Blood sample centrifugation was conducted at 3000 rpm for 15 minutes to get serum samples stored at −20°C for the colorimetric assay according to the manufacturer's protocol, as described by Mousa and Aldebasi [39].

### 2.9. Statistical Analysis of Data

The mean (M) ± standard deviation (SD) of the data was analyzed statistically by the SPSS software program (IBM, USA), and the one-way ANOVA test followed by LSD was performed to evaluate the intergroup comparisons. At first, the normality tests (skewness and Kurtosis) were performed and revealed the normal data distribution. *P* ≤ 0.05 indicated statistically significant results. The GraphPad Prism software version 6.0 (GraphPad Software Inc., San Diego, CA) was used to create graphs of the analyzed data [12, 40].

## 3. Results

### 3.1. CPMCSO and CPMMH Effects on the Macroscopic Hematuria Scale of UB

**Figure 2:**
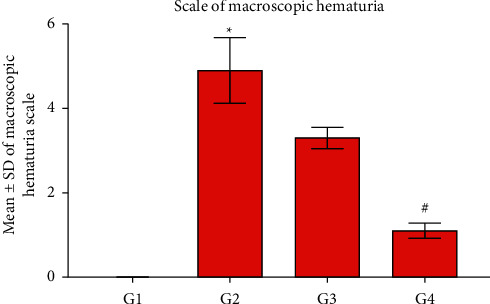
Statistical analysis of the macroscopic hematuria scale. The CP group (G2) exhibits a significant elevation of the hematuria scale compared with the control group (G1) and the CPMMH regimen group (G4). In contrast, G4 reveals a significant reduction of the hematuria scale compared with the CPMCSO regimen group (G3). ^*∗*^*P* ≤ 0.05 vs. G1 and G4 and ^#^*P* ≤ 0.05 vs. G3.

Figure 2 exhibited a significant (*P* ≤ 0.05) elevation of the hematuria scale in G2 compared with G1 and G4 rabbits. In contrast, G4 revealed a significant (*P* ≤ 0.05) reduction of the hematuria scale compared with G3 rabbits.

### 3.2. CPMCSO and CPMMH Effects on the UB Structure

**Figure 3:**
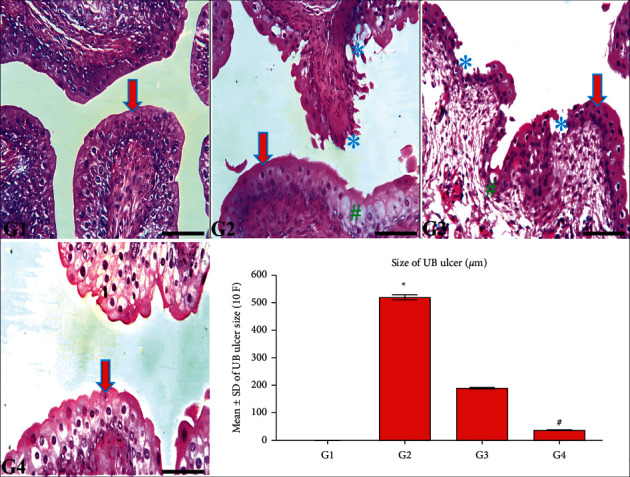
Histological structure of the urinary bladder (UB) in all groups. The UB of G1 and G4 rabbits show typical urothelial structure (arrow) compared with the marked urothelial vacuolar degeneration (^#^) and ulceration (^*∗*^) in G2 and G3. In contrast, G4 reveals a significant improvement of urothelial ulceration compared with G3. H&E; 200x, bar = 100 *µ*m. Statistical analysis of the UB ulcer's size/10 fields (F). ^*∗*^*P* ≤ 0.05 vs. G1 and G4 and ^#^*P* ≤ 0.05 vs. G3.

Figure 3 revealed a typical structure of the UB in G1 and G4 rabbits, elucidating a wholly protective role of the CPMMH regimen against HC in G4. In contrast, G2 exhibited an obvious abnormality of UB structure (urothelial vacuolar degeneration, ulceration, and sloughing) compared with G1, elucidating the harmful effects of CP on the UB of G2 rabbits. Besides, G3 revealed mild erosion and ulceration of the urothelium compared with G2, elucidating the partial protective role of the CPMCSO regimen against HC in G3 rabbits. Statistical analysis of the urothelial ulcer's size confirmed the appearance of HC in G2 compared with G1 and G4 rabbits.

### 3.3. CPMCSO and CPMMH Effects on CF Deposition in the UB

**Figure 4:**
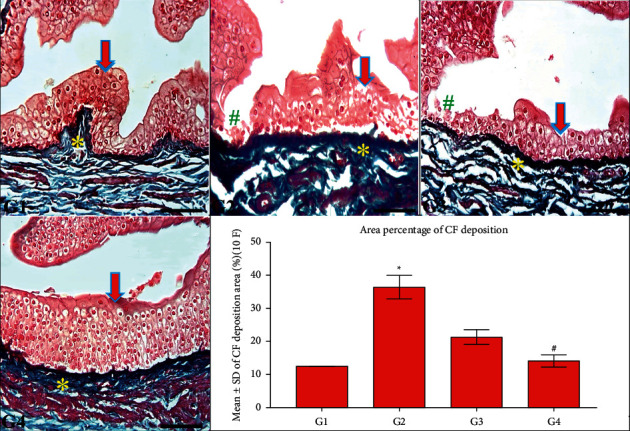
Collagen fibers (CF) deposition in the UB of all groups. G1 and G4 rabbits exhibit normal urothelial structure (arrow) and mild CF deposition (^*∗*^) in the UB. In contrast, the UB wall shows urothelial ulceration (^#^) and moderate CF deposition (^*∗*^) in G2 and G3 compared with G4 rabbits. Masson's trichrome; 200x, bar = 100 *µ*m. UB statistical analysis of CF deposition/10 (F) ^*∗*^*P* ≤ 0.05 vs. G1 and G4 and ^#^*P* ≤ 0.05 vs. G3.

Figure 4 exhibited average deposition of CF in G1 and G4 rabbits, indicating the protective role of the CPMMH regimen against HC and UB fibrosis in G4. In contrast, G2 and G3 rabbits revealed urothelial ulceration and moderate CF deposition in the UB, indicating substantial UB fibrosis in both groups.

### 3.4. CPMCSO and CPMMH Effects on TNF-*α* IE in the UB

**Figure 5:**
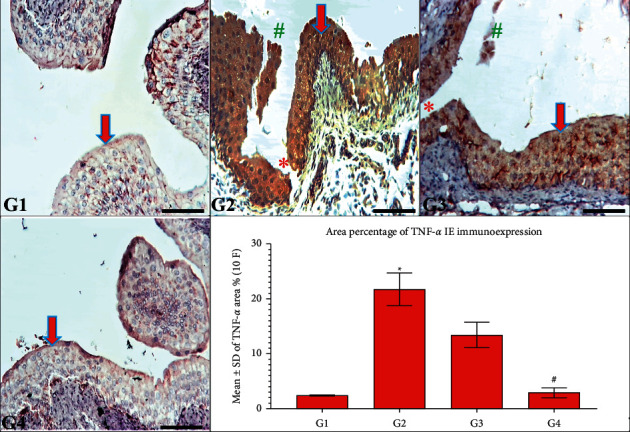
TNF-*α* immunoexpression in the UB of all groups. G1 and G4 rabbits show typical UB structure and mild TNF-*α* IE (arrow). In contrast, G2 and G3 rabbits show urothelial ulceration (^*∗*^) and sloughing (^#^) with marked TNF-*α* IE (arrow) compared with G4 rabbits. TNF-*α* immunostain; 200x, bar = 100 *µ*m. Statistical analysis of TNF-*α* IE/10 (F) ^*∗*^*P* ≤ 0.05 vs. G1 and G4 and ^#^*P* ≤ 0.05 vs. G3.

Figure 5 exhibited mild TNF-*α* IE in the UB of G1 and G4 rabbits, elucidating complete anti-inflammatory protection against HC by the CPMMH regimen in G4. In contrast, G2 and G3 revealed marked urothelial TNF-*α* IE, indicating a weak anti-inflammatory protective effect of the CPMCSO regimen on the UB of G3 rabbits.

### 3.5. CPMCSO and CPMMH Effects on the Urothelial Ultrastructure

**Figure 6:**
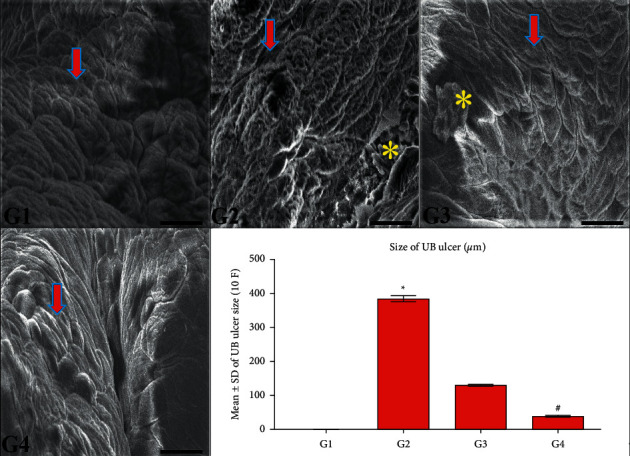
Ultrastructure of the urinary bladder's (UB) urothelial surface in all groups. G1 and G4 show a standard urothelial surface structure (arrow). In contrast, the area percentage of urothelial ulcer (^*∗*^) significantly increases in G2 compared with G1 and G4. Besides, the urothelial ulcer significantly decreases in G4 compared with G3 (^*∗*^). SEM; 15 kv, 1000×, bar = 20 *µ*m. Statistical analysis of the area percentage of urothelial ulcers. ^*∗*^*P* ≤ 0.05 vs. G1 and G4 and ^#^*P* ≤ 0.05 vs. G3.

SEM examination revealed the typical urothelial structure of G1 and G4 in Figure 6, indicating wholly urothelial protection by the CPMMH regimen in G4. In contrast, the urothelial ulcers were significantly increased in G2 (confirming urothelial ulceration) compared with G4 and significantly reduced in G4 compared with G3 (elucidating a partial protective role of the CPMCSO regimen on the urothelium of G3).

### 3.6. CPMCSO and CPMMH Effects on the Activity of Antioxidant Enzymes

**Figure 7:**
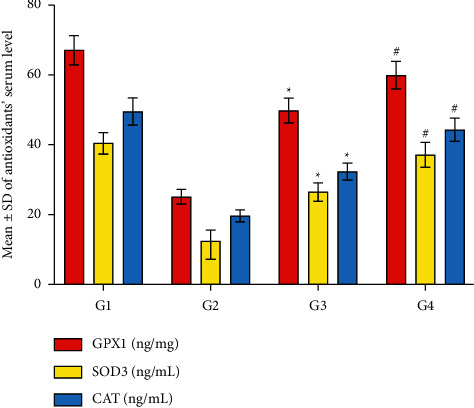
Statistical analysis of the antioxidant enzymes (GPx1, SOD3, and CAT) activity. G2 shows significant reductions in the antioxidant activity compared with G1 and G4. In contrast, the antioxidant activity shows significant elevation in G4 compared with G3. ^*∗*^*P* ≤ 0.05 vs. G1 and G4 and ^#^*P* ≤ 0.05 vs. G3.

Figure 7 revealed significant reductions in the activity of antioxidant enzymes (GPx1, SOD3, and CAT), indicating a marked induction of oxidative stress by CP administration in G2 compared with G1 and G4. In contrast, the antioxidant activity significantly increases by the CPMMH regimen in G4 compared with G3, indicating the potent antioxidant activity of the CPMMH regimen against HC in G4 compared with the moderate antioxidant activity of the CPMMH regimen in G3.

### 3.7. CPMCSO and CPMMH Effects on the Proinflammatory Cytokines

**Figure 8:**
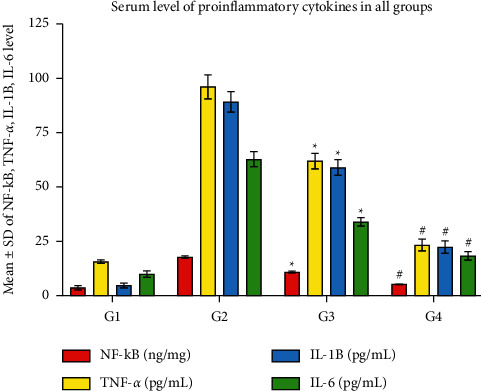
Statistical analysis of the proinflammatory cytokines (NF-*κ*B, TNF-*α*, IL-1B, and IL-6). G2 shows significant elevations of the proinflammatory cytokines' serum levels compared with G1 and G4. In contrast, G4 reveals significant reductions of the proinflammatory cytokines' levels compared with G3. ^*∗*^*P* ≤ 0.05 vs. G1 and G4 and ^#^*P* ≤ 0.05 vs. G3.

Figure 8 reveals significantly elevated serum levels of proinflammatory cytokines (NF-kB, TNF-*α*, IL-1B, and IL-6) in G2 compared with G1 and G4, indicating the extensive induction of proinflammatory cytokines' production by CP administration in G2. In contrast, the CPMMH regimen caused marked reductions of the proinflammatory cytokines' levels, indicating its substantial anti-inflammatory effects on G4 compared with G3 rabbits.

## 4. Discussion

It is evident that CP-induced HC is mainly sparked by renal excretion of acrolein, which induces inflammatory reactions in the UB by enhancing the oxidative stress process and releasing the proinflammatory cytokines [28]. Therefore, finding a novel, safe, and selective therapeutic modality rich in antioxidants and anti-inflammatory agents is expected to be an enduring progression for avoiding HC. The CPMCSO and CPMMH regimens involve the coadministration of CP plus mesna and CSO or mesna and MH. Both regimens are rich in antioxidants and anti-inflammatory agents (bioactive flavonoids and polyphenols) [41].

Rabbits of the current study were treated with the CPMCSO or CPMMH regimen to determine which one of them is more protective against CP-induced HC. The main HP, IHC, and SEM features of HC appeared as urothelial degeneration, ulceration, and sloughing in G2, which were significantly attenuated with remarkable curative effects on HC by the CPMMH regimen in G4 compared with the CPMCSO regimen in G3 rabbits. Additionally, it was not surprising that CP induced a significant reduction of the antioxidant enzymes (GPx1, SOD3, and CAT) activity and significantly elevated the levels of proinflammatory cytokines (NF-*κ*B, TNF-*α*, IL-1, and IL-6) in G2 compared with G1 and G4 rabbits. In contrast, the CPMMH regimen significantly elevated the antioxidants activity and significantly reduced the levels of proinflammatory cytokines in G4 compared with the CPMCSO regimen in G3 rabbits.

The pathogenesis of CP-induced HC could be explained by several studies, which reported that toxic acrolein metabolites directly contact the urothelium, induce the transcription of NF-*κ*B factor, and activate the overproduction of intracellular ROS and RNS, leading to the marked formation of ONOO^−^ [42, 43]. Besides, these substances influence the progression of HC by inducing lipid peroxidation, depleting numerous cellular proteins, and evoking the cascade of cellular proinflammatory mediators, with subsequent UB injury [44, 45]. Moreover, NO and ONOO^−^ activation disrupts the UB integrity, exaggerates the oxidative stress process, and activates the inflammatory cells (especially macrophages), leading to potentiation of NF-*κ*B expression and overproduction of the proinflammatory cytokines TNF-*α*, IL-1B, and IL-6 [46, 47].

On the other hand, mesna is transformed into dimesna, which is rapidly excreted in urine to create a nontoxic, inert dimer (thioether) on the mucosal surface of UB by direct coupling of dimesna with acrolein [48]. Lack of mesna antioxidant and anti-inflammatory activity limits its effectiveness, leading to ineffective prevention of HC in 25% of cases treated with CP.

Nowadays, numerous studies have proved that dietary modifications could become an essential adjuvant therapeutic line in minimizing UB damage by enhancing the antioxidative defense system, augmenting the anti-inflammatory effects, and abolishing the severity of UB damage [49]. Hence, numerous natural products such as celery and honey could be safe, effective, and cheap agents against HC due to their antioxidant and anti-inflammatory effects [50]. Therefore, a real need to coadministrate extra natural antioxidants and anti-inflammatory agents with mesna exists to enhance its protective role against HC. In the current work, we expected that coadministration of mesna with these agents could be a novel effective chemotherapeutic regimen for protection against CP-induced HC [51].

Indeed, the CSO has several active constituents with potent antioxidant and anti-inflammatory properties, including d‐limonene, sedanolide, terpenoids, polyphenols, apiin, and apiuman [52, 53]. Therefore, the potential effects of these compounds improved the healing power of urothelium, enhanced the activity of antioxidant enzymes (GPx1, SOD3, and CAT), and subsequently improved the CPMCSO regimen's efficacy against HC in G3 rabbits [14]. Besides, CSO has been ameliorated the damaged UB tissues via enhancing the scavenging power of free radicals and reducing the lipids peroxidation process [54, 55]. Additionally, the anti-inflammatory properties of CSO could be attributed to the inhibitory effects of apiin and apiuman against the expression of proinflammatory cytokines (NF-*κ*B, TNF-*α*, IL-1B, and IL-6) [16].

On the other hand, several studies explained the healing power of MH via its various antioxidant, antimicrobial, and anti-inflammatory effects on the inflammatory response [56, 57]. The potent antioxidant capacity of MH in G4 rabbits could be attributed to its high contents of phenolic compounds, which modulate the free radical production and protect the cell components from the harmful effects of ROS [58]. At the same time, the diverse phenolic compounds and defensin-1 in MH may account for its potential antimicrobial effects [59]. Furthermore, the potent protective anti-inflammatory mechanisms of MH could be explained via the suppression of inflammatory cells migration at the wound site, the reduction of proinflammatory cytokines (TNF-*α*, IL-1*β*, and IL-6) production, and the enhancement of fibroblasts proliferation, which improves the wound healing process [60].

To sum up, the CPMMH regimen significantly improves the antioxidants activity, reduces the NF-*κ*B, TNF-*α*, IL-1*β*, and IL-6 activation, and ensures better protection of the UB in G4 rabbits compared with the partial protection against UB toxicity by the CPMCSO regimen in G3 rabbits.

## 5. Conclusion

The current study concludes that CP therapy induced apparent hazardous urothelial oxidative stress and impaired the healing process of UB, which developed HC and remarkably impeded the usage of CP against several neoplastic diseases. In contrast, the CPMMH regimen revealed marked improvement of the UB structure and caused lesser inflammation and ulceration of the urothelium. Additionally, it significantly improves the antioxidant activity of GPx1, SOD3, and CAT enzymes, reduces the NF-*κ*B, TNF-*α*, IL-1B, and IL-6 cytokines activation, and ensures better protection of the UB in G4 rabbits compared with the partial protection against UB injury by the CPMCSO regimen in G3. Therefore, the CPMMH regimen seems to be more effective than the CPMCSO regimen for combating HC and could be a novel future cotherapy against CP-induced HC.

## Data Availability

All relevant data have been provided in the manuscript and supplementary material.

## Supplementary Materials

Supplementary Materials The supplementary file includes highlights and a graphical abstract of the study.
