# Supplementary material for: Cytoprotective Antioxidant, Anti-Inflammatory, and Antifibrotic Impact of Celery Seed Oil and Manuka Honey Against Cyclophosphamide-Induced Cystitis in Rabbits
Source: Evid Based Complement Alternat Med. 2022 Mar 17;2022:2863023. doi: 10.1155/2022/2863023 (PMC8947928; doi:10.1155/2022/2863023)

Supplementary file

Highlights

- Cyclophosphamide-induced hemorrhagic cystitis (HC) in rabbits.
- Mesna + manuka honey (MMH) decreased urinary bladder inflammation and fibrosis.
- MMH regimen increased antioxidant activity of GPx1, SOD3, and CAT enzymes
- MMH regimen decreased proinflammatory cytokines (NF-κB, TNF-α, IL-1B, and IL-6).
- MMH regimen could be an effective therapy against cyclophosphamide-induced HC

Graphical abstract

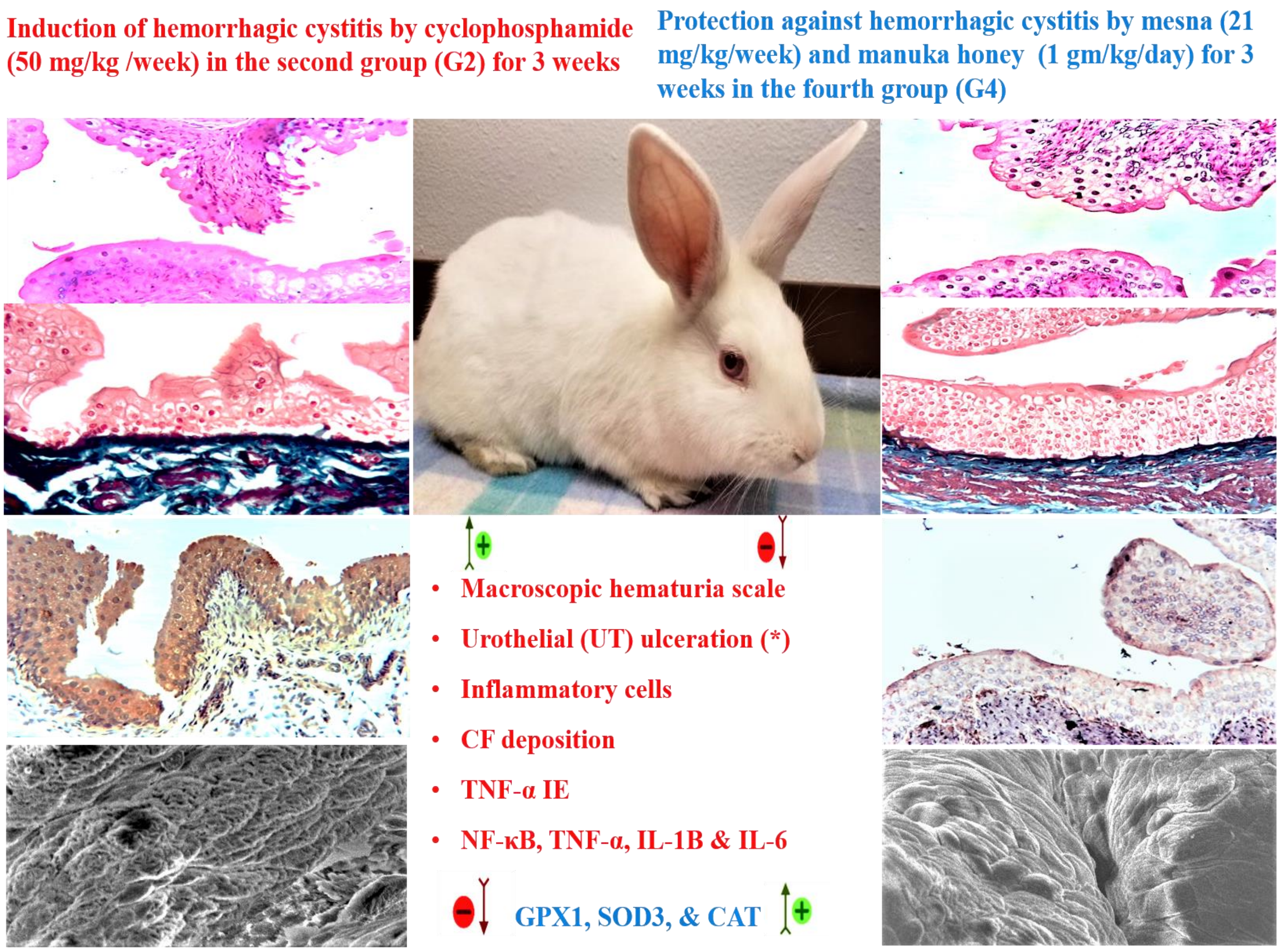

Supplement: Supplementary Materials — The supplementary file includes highlights and a graphical abstract of the study. [file 2863023.f1.pdf]
